# Ethnobotanical Study of Plants Used in the Management of HIV/AIDS-Related Diseases in Livingstone, Southern Province, Zambia

**DOI:** 10.1155/2016/4238625

**Published:** 2016-03-16

**Authors:** Kazhila C. Chinsembu

**Affiliations:** Faculty of Science, Department of Biological Sciences, University of Namibia, Private Bag 13301, Windhoek, Namibia

## Abstract

Faced with critical shortages of staff, long queues, and stigma at public health facilities in Livingstone, Zambia, persons who suffer from HIV/AIDS-related diseases use medicinal plants to manage skin infections, diarrhoea, sexually transmitted infections, tuberculosis, cough, malaria, and oral infections. In all, 94 medicinal plant species were used to manage HIV/AIDS-related diseases. Most remedies are prepared from plants of various families such as Combretaceae, Euphorbiaceae, Fabaceae, and Lamiaceae. More than two-thirds of the plants (mostly leaves and roots) are utilized to treat two or more diseases related to HIV infection. Eighteen plants, namely,* Achyranthes aspera* L.,* Lannea discolor* (Sond.) Engl.,* Hyphaene petersiana* Klotzsch ex Mart.,* Asparagus racemosus* Willd.,* Capparis tomentosa* Lam.,* Cleome hirta* Oliv.,* Garcinia livingstonei* T. Anderson,* Euclea divinorum* Hiern,* Bridelia cathartica* G. Bertol.,* Acacia nilotica* Delile,* Piliostigma thonningii* (Schumach.) Milne-Redh.,* Dichrostachys cinerea* (L.) Wight and Arn.,* Abrus precatorius* L.,* Hoslundia opposita* Vahl.,* Clerodendrum capitatum* (Willd.) Schumach.,* Ficus sycomorus* L.,* Ximenia americana* L., and* Ziziphus mucronata* Willd., were used to treat four or more disease conditions. About 31% of the plants in this study were administered as monotherapies. Multiuse medicinal plants may contain broad-spectrum antimicrobial agents. However, since widely used plants easily succumb to the threats of overharvesting, they need special protocols and guidelines for their genetic conservation. There is still need to confirm the antimicrobial efficacies, pharmacological parameters, cytotoxicity, and active chemical ingredients of the discovered plants.

## 1. Introduction

Livingstone has the highest human immunodeficiency virus (HIV) prevalence level in Zambia. Although the average HIV prevalence rate in Zambia is about 13%, the HIV infection rate in Livingstone is about 25.3%, significantly higher than the national average [1]. During the period 1994–2002, Livingstone's HIV prevalence was stable at around 30% [2]. Located in Southern Province, Livingstone is the tourist capital of Zambia, home to the famous Victoria Falls. Therefore, many socioeconomic factors conspire to fuel the town's growing HIV epidemic. Transactional sex is very common in Livingstone, attributable to local or foreign tourists and high levels of poverty; receiving money or gifts for sex is the only means for vulnerable women to financially secure themselves and their families because other sources of income are not sufficient [3].

According to Byron et al. [4], women combine sex with the sale of material products to earn higher profits when they go to the market in town and end up contracting HIV. At 3.1%, women in Livingstone had the highest prevalence of sexually transmitted infections (STIs) in Zambia [1]. Migrant labourers, especially sugar cane cutters from Mazabuka (Zambian town with the second highest HIV prevalence at 18.4%), are often blamed for being high-risk transmitters of HIV. In several sites in Livingstone, there are designated venues where people meet new sexual partners [2]. These sexual venues are linked to high partner turnover and due to major challenges in on-site condom availability, unprotected sex is common among guests [2]. Livingstone also has low rates of male circumcision at 11% [1].

In Livingstone, studies have reported sexual behaviours with a high potential for HIV transmission, yet there are few signs of HIV preventive interventions [3]. Despite the rollout of antiretroviral therapy (ART), Cataldo and others [5] stated that Zambian HIV-infected persons still seek treatment from traditional healers. Thus, although some western trained health care providers remain suspicious of traditional healers, most agree that traditional healers play an important and complementary role in the provision of effective HIV prevention or treatment [6]. Kaboru [7] also found that many biomedical health practitioners believe that Zambian traditional healers can help control HIV/AIDS.

Undoubtedly, several patients seek herbal remedies for conditions related to acquired immune deficiency syndrome (AIDS) before seeking care at health centres [8]. This is because there are many deficiencies in the provision of biomedical services for STIs and HIV/AIDS in Zambia [7]. Unlike hospital staff with poor attitudes, traditional healers are also kinder and more compassionate to patients [7]. According to Ndulo et al. [9], traditional healers attend to patients with sexually transmitted infections (STIs), in both rural and urban areas; therefore, efforts should be made to promote cooperation between traditional and biomedical health care providers, so that treatment of patients and their partners could be improved. Traditional management that concurs with biomedical practices could thus be a starting point for discussion and cooperation.

Moreover, traditional healers have good knowledge of STIs [9]. Most of them use herbal preparations in the form of roots or powders administered orally to induce diarrhoea, vomiting, and diuresis. Traditional healers also correctly cite symptoms associated with STIs such as urethral or vaginal discharge. Therefore, Makasa et al. [10] observed that about 15% of patients with genital ulcer disease seek treatment from traditional healers. Although the use of traditional medicine is associated with nonadherence to ART, health care providers at hospitals should open lines of communication with traditional healers [11]. Makasa et al. [10] also noted the need to increase awareness among traditional healers that handle patients presenting with STIs and to refer certain cases to health facilities, especially when patients do not respond to traditional medicines.

Traditional healers far outnumber modern health care providers in Zambia where the Traditional Health Practitioners Association has over 40,000 members compared to a paltry 1,000 conventional medical doctors that are practicing nationwide [11]. There were 1,390 medical doctors practicing in Zambia; the doctor to population ratio was 1 to 17,589 instead of the World Health Organization recommended ration of 1 to 5,000 [12]. At the entrance of Zambia's University Teaching Hospital (UTH), a signpost reads “kindly take note that members of staff at UTH work under very critical shortage of manpower,” a stark reminder of the dearth of health staff and severe crisis facing the country. Given the glaring personnel shortages in many public health care facilities [13], involvement of traditional healers in the management of HIV/AIDS opportunistic diseases is a ubiquitous narrative.

Zambia is among the Sub-Saharan African countries with the most acute shortages of trained personnel in the health sector [14]. Predictably, use of traditional medicines was reported among 75% of inpatients at the UTH and among 68% of those seeking services for HIV counselling and testing [11]. Studies have shown that individuals that use traditional medicines are also associated with alcohol, have two or more sexual partners, engage in dry sex, and harbour STIs. Corollary, identification of persons who access traditional medicines may be an important target population for HIV prevention because many HIV risky behaviours are common among clients of traditional healers [11].

Besides, traditional healers are still consulted because they are deemed to provide client-centred and personalised health care that is customized to the needs and expectations of patients, paying special respect to social and spiritual matters [15]. Indeed, whilst the majority of HIV/AIDS patients that need treatment can access ART from local hospitals and health centres, several constraints of the ART programme compel many HIV-infected people to use traditional medicines to manage HIV/AIDS-related conditions [16]. Others use ethnomedicinal plants to offset side effects from ART [17]. The use of medicinal plants in Livingstone is also part of the medical pluralism whereby the introduction of allopathic medicines has not really dampened beliefs in indigenous diagnosis and therapeutic systems [18].

Even though there are some anecdotal reports regarding the traditional uses of ethnomedicinal plants to manage various diseases in Livingstone, knowledge on specific plant species used to manage HIV/AIDS-related diseases is still scanty and not well recorded. This paper is an inaugural and modest report on medicinal plants used in the management of HIV/AIDS opportunistic infections in Livingstone, Southern Province, Zambia. Documentation of putative anti-HIV plant species may help preserve this critical tacit indigenous knowledge resource. Plus, indigenous knowledge, coupled with a history of safe use and ethnopharmacological efficacy of medicinal flora, also presents a faster approach to discover new chemical compounds that may be developed into novel antiretroviral drugs.

## 2. Materials and Methods

### 2.1. Study Area

**Figure 1:**
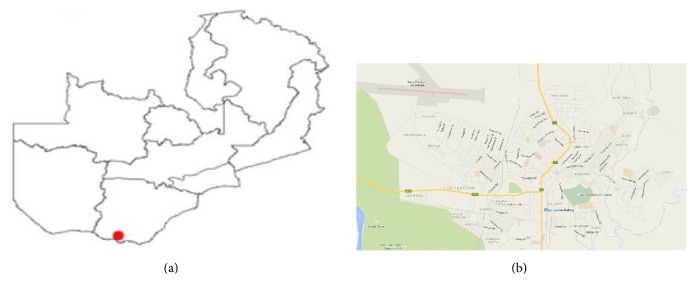
(a) Map of Zambia showing the location of Livingstone. (b) Townships in Livingstone town.

The study was carried out in Chibelenga, Burton, Dambwa, Hillcrest, Libuyu, Linda, Malota, Maramba, Ngwenya, and Zakeyo; these form the urban and rural settlements of Livingstone, provincial headquarters of Southern Province until 2012 (Figure 1). The geographical coordinates of Livingstone are 17°51′0′′ south, 25°52′0′′ east. The town is situated about 981 metres above sea level near the Victoria Falls on the Zambezi River close to the Zimbabwean border. Situated in agroecological region I, Livingstone has a humid subtropical climate. Its average annual rainfall is about 690–740 mm. The mean maximum temperature is high, 35°C in October, and the mean minimum temperature is low, 7°C in June. Recorded high (41.1°C) and low (−3.7°C) temperatures in November and June, respectively, have been documented. Rainy season occurs between November and March when it is wet, hot, and humid.

According to the Holdridge life zones system of bioclimatic classification, Livingstone is situated in or near the subtropical dry forest biome. The terrain in Livingstone is well vegetated with over 1,000 plant species represented by riparian forests and woodlands, Kalahari woodlands,* Colophospermum mopane* (J. Kirk ex Benth.) J. Léonard woodlands, and deciduous woodlands mostly consisting of* Brachystegia glaucescens* Hutch. and Burtt Davy and the tall mahogany* Entandrophragma caudatum *Sprague. The vegetation is quite similar to that in adjoining Sesheke District as reported by Chinsembu [18] except for a few dominant plant species.

Between 1907 and 1935, Livingstone was the capital city of Northern Rhodesia, now Zambia. The town is named after David Livingstone who in 1840 as a young Scottish doctor and ordained minister sailed from Britain to the Cape to work as a medical evangelist with the London Missionary Society. In 1855, Dr. David Livingstone became the first European to see the Victoria Falls when he was taken there by Sekeletu, chief of the Subiya/Kololo people. Although contemporary life is a blend of values and traditions of more than 70 of Zambia's ethnically diverse people, the main tribes in Livingstone are the Tonga/Tokaleya and Lozi; many of them live in townships such as Maramba. Archaeological artifacts suggest the existence of the Tonga for at least 900 years in southern Zambia's Zambezi Valley. The Lozi migrated into Western Zambia from the Luba/Lunda Kingdom of Mwata Yamvwa in Zaire, present day Democratic Republic of the Congo.

After Zambia's independence in 1964, President Kenneth Kaunda's government built motor vehicle and radio assembly plants in Livingstone, attracting migrant workers. These manufacturing industries closed soon after President Frederick Chiluba became president of Zambia in 1991. In recent years, the town's economic fortunes have dwindled except for a slight influx of investment in the tourism sector characterized by the opening of modern hotel chains like Sun International. Commercial sex work is common among women; many of them are from neighbouring Zimbabwe where the socioeconomic situation remains dire.

### 2.2. Ethnobotanical Data Collection

Ethnobotanical data were collected using methods similar to that of [17–19]. Briefly, snowball sampling was applied during ethnobotanical surveys of thirty knowledge holders including 10 traditional healers that use plants to treat HIV/AIDS-related diseases. Before conducting interviews, the aim of the study was clearly explained and knowledge holders were asked for their consent. Then the knowledge holders were individually engaged in semistructured interviews supplemented with questionnaires. During the conversations, data on respondent characteristics and information related to medicinal uses of plants for the management of HIV/AIDS-related diseases were captured. All interviews were conducted in local languages, Tonga/Tokaleya, and Lozi. Research assistants acted as Tonga/Tokaleya/Lozi to English translators.

Data were collected during two stages consisting of primary and secondary samplings. The primary stage involved an exploratory and descriptive study of eight knowledge holders that manage HIV/AIDS-related infections. The focus of the exploratory study was to gain critical insights into the work of the knowledge holders, distil pertinent issues, and gauge whether a detailed ethnobotanical survey would be feasible. Knowledge holders were asked about the main symptoms of HIV/AIDS, their healing practices, and sources of ethnomedicinal knowledge. The following data in relation to the plants were also recorded: vernacular names (Tonga/Tokaleya/Lozi), plant habits, plant parts used, the HIV/AIDS-related conditions treated with the plants, and the modes of preparation and application of the plant remedies to the patient.

The secondary sampling stage was a follow-up and detailed descriptive study of 22 knowledge holders who verified prior ethnobotanical data obtained from others during the exploratory inquiry. To allow for triangulation of ethnomedicinal use, only plants mentioned by at least three knowledge holders in the descriptive study (for each disease condition) were eligible for documentation [20]. On-the-spot identification of familiar plant species was done in the field. Voucher numbers for plants were assigned and specimens for plants were collected in herbarium plant presses for identification and confirmation. Botanical names were verified using the International Plant Name Index (IPNI).

### 2.3. Data Analysis

Quantitative analysis of ethnobotanical data was done by calculating percentage frequencies, familiarity index *F*_*i*_, and factor informant consensus (*F*_IC_). The *F*_*i*_, a relative indicator of the familiarity of a plant species, is defined as the frequency a given plant species is mentioned as an ethnomedicine divided by the total number of knowledge holders interviewed in the study [21]. The *F*_*i*_ was calculated as follows:

(1) $$\begin{array}{c} F_{i}=\frac{N_{a}}{N_{b}}\times 100, \end{array}$$

where *N*_*a*_ is the number of informants that mention a species as a medicine and *N*_*b*_ is the total number of respondents.

The *F*_IC_ was the number of use citations in each ailment category (*N*_ur_) minus the number of species used (*N*_*t*_), divided by the number of use citations in each category minus one [22]:

(2) $$\begin{array}{c} F_{IC}=N_{ur}-\frac{N_{t}}{\left(N_{ur}-1\right)}. \end{array}$$

*F*_IC_ values are low (near 0) if plants are chosen randomly or if informants do not exchange information about their use. Values are high (near 1) if there was a well-defined selection criterion among informants and/or if information was exchanged between informants. High *F*_IC_ values are also obtained when only one or a few plant species are reportedly used by a high number of knowledge holders to treat a particular disease, and low *F*_IC_ values imply that respondents disagree over which plant to use [23].

## 3. Results

Of all the thirty knowledge holders included in the study, only eight were female. This gender difference may be explained by the fact that male knowledge holders in the community were more comfortable to talk about STIs than female knowledge holders who face cultural restrictions when it comes to talking about matters related to sex, STIs, and HIV/AIDS. The average age of the healers was 48 years. About 70% of the knowledge holders received their medicinal plant knowledge from their older family members and the remainder from spiritual and supernatural powers such as ancestral spirits, dreams, and visions. Only six traditional healers had an apprentice under their tutelage; the rest did not train other people.

Medicobotanical data including the plants' scientific names, vernacular names, families, voucher numbers, habits, frequency indices, parts, HIV/AIDS-related diseases treated, modes of preparation, and application are described in Table 1. Overall, 94 plant species from 39 families were used by various knowledge holders to manage HIV/AIDS-related diseases in Livingstone, Southern Province, Zambia (Table 1). Their growth habits were as follows: almost half were trees (53.2%), about a quarter were shrubs (24.5%), and there were approximately equal proportions of climbers (11.7%) and herbs (10.6%).

**Figure 2:**
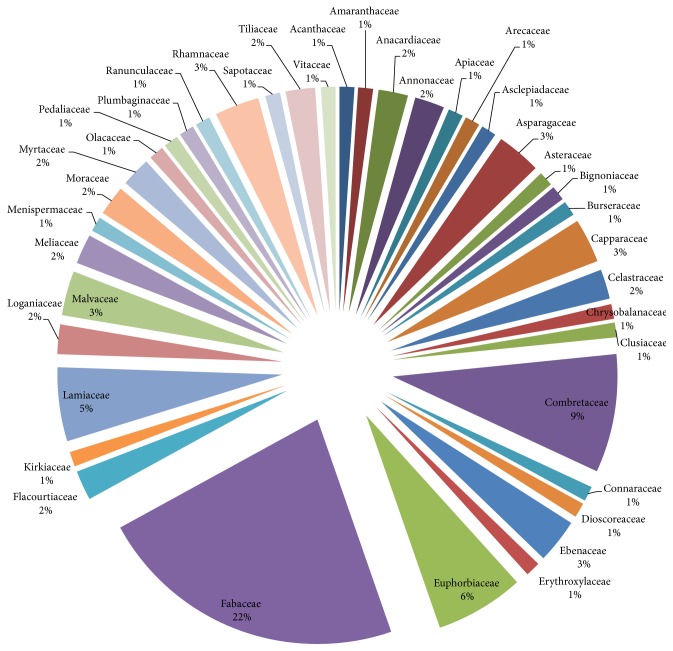
Percentage use of plant families.

**Figure 3:**
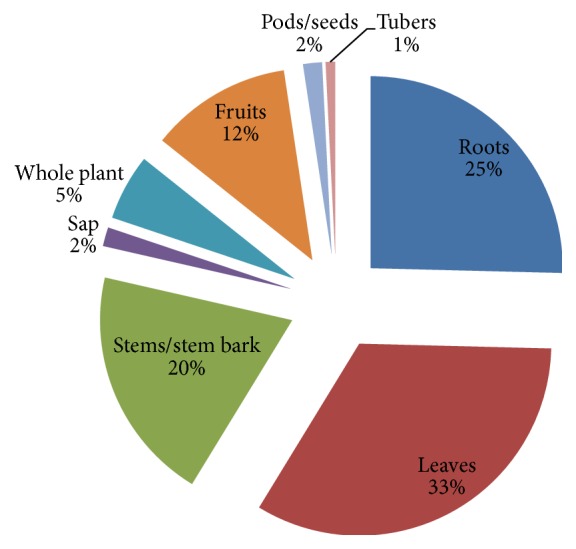
Percentage use of plant parts.

**Figure 4:**
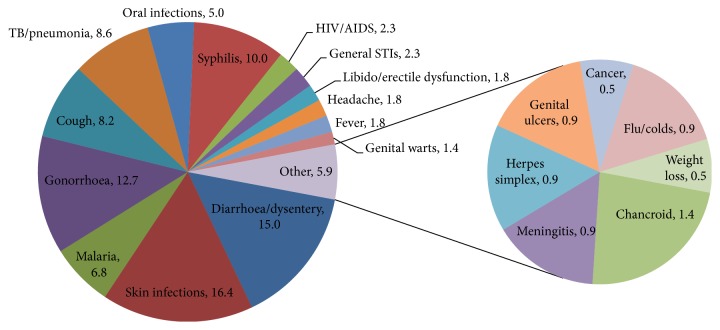
Proportions of plants used to treat different disease conditions.

The most used families were Fabaceae (22%), Combretaceae (9%), Euphorbiaceae (6%), and Lamiaceae (5%) (Figure 2). The most plant parts used were leaves (33%), roots (25%), bark (22%), and stems/stem barks (20%) (Figure 3). Pods/seeds (2%) and tubers (1%) were least used. Plant exudates in the form of sap were also harvested from 2% of the plants.  Figure 4 presents the proportions of plant species used to treat various HIV/AIDS-related disease conditions: skin infections (16.4%), diarrhoea/dysentery (15.0%), gonorrhoea (12.7%), syphilis (10.0%), tuberculosis (TB)/pneumonia (8.6%), cough (8.2%), malaria (6.8%), and oral infections (5.0%).

**Figure 5:**
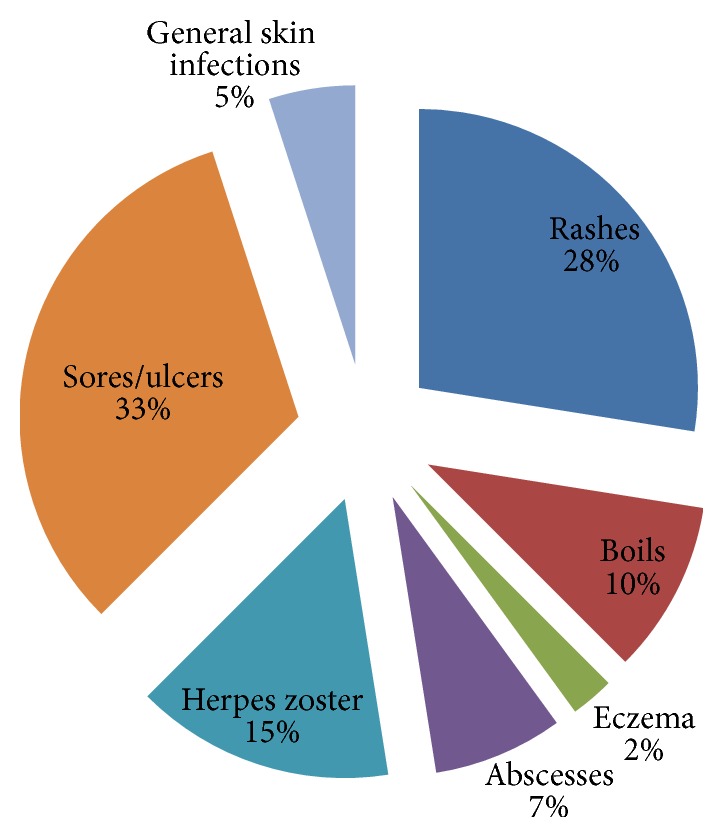
Percentage distribution of plants used to treat various skin conditions.

**Figure 6:**
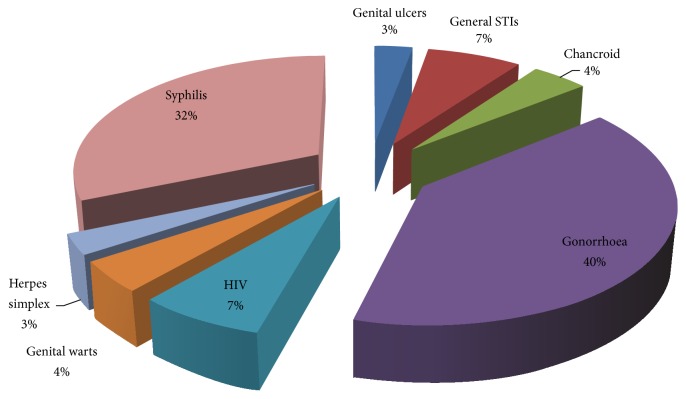
Percentage distribution of plants used to treat various STIs.

Figure 5 illustrates that of all the plants that were used to ameliorate skin conditions, most of them were used to manage skin sores or ulcers (33.0%), rashes (28.0%), herpes zoster (15.0%), boils (10.0%), and abscesses (7.0%). About 5% of all plants used on skin conditions treated general infections. Of all the ethnomedicinal plants used to manage STIs, the majority of them were used for gonorrhoea (40.0%), syphilis (32.0%), and HIV (7.0%) (Figure 6).

Eighteen plants were utilized to treat four or more disease conditions:* Achyranthes aspera* L.,* Lannea discolor* (Sond.) Engl.,* Hyphaene petersiana* Klotzsch ex Mart.,* Asparagus racemosus* Willd.,* Capparis tomentosa* Lam.,* Cleome hirta* Oliv.,* Garcinia livingstonei* T. Anderson,* Euclea divinorum* Hiern,* Bridelia cathartica* G. Bertol.,* Acacia nilotica* Delile,* Piliostigma thonningii* (Schumach.) Milne-Redh.,* Dichrostachys cinerea* (L.) Wight and Arn.,* Abrus precatorius* L.,* Hoslundia opposita* Vahl,* Clerodendrum capitatum* (Willd.) Schumach.,* Ficus sycomorus* L.,* Ximenia americana* L., and* Ziziphus mucronata* Willd. Only 31% of the plants in this study were administered as monotherapies.

The *F*_*i*_ values are given in Table 1. Informants were more familiar with the medicinal uses of the following fourteen most frequently used plants:* Cassia abbreviata* Oliv.,* Combretum imberbe* Wawra,* Diospyros mespiliformis* Hochst. ex A.DC.,* Fockea angustifolia *K. Schum.,* G. livingstonei*,* Kigelia africana* (Lam.) Benth.,* Mimosa pigra* L.,* Syzygium cordatum* Hochst.,* Syzygium guineense* DC.,* Terminalia prunioides* M. A. Lawson,* Peltophorum africanum* Sond.,* Plumbago zeylanica* L.,* X. americana*, and* Z. mucronata*. According to Table 2, *F*_IC_ values for the various disease conditions show that consensus was high over plants used to treat malaria, oral infections, and fever/flu/colds/headache.

## 4. Discussion

The highest proportion of plants in Livingstone was used to manage skin diseases, probably because they contain antimicrobial agents. A similar scenario was obtained in Sesheke (Zambia) and Rundu (Namibia). This speaks to the fact that skin infections are quite common during HIV infection. Many of the plants for skin diseases in Livingstone were used to manage skin infections in other geographical settings. For instance, Afolayan et al. [24] and Hedimbi and Chinsembu [25] documented the use of* Asparagus* species in the treatment of eczema in South Africa and Namibia, respectively.* Friesodielsia obovata* was used to treat skin infections in the Zambezi Region of Namibia [26].

*Capparis tomentosa *is also used to treat skin rashes and herpes zoster in Katima Mulilo, Namibia [17]. Many* Acacia* species are also used to manage skin conditions in Southern Africa [27]. Kenyans use* Trichilia emetica* and* Syzygium guineense* to treat skin cancers [28]. Leaves of one of the fig trees,* Ficus capensis,* were also a remedy for skin sores. Skin diseases lie at the centre of both Christian and Islamic faiths. Indeed, the use of figs to treat skin diseases such as boils is well documented in the Bible; see 2 Kings 20:7 where a poultice of common figs (*Ficus* sp.) was applied to heal boils.

*Lannea stuhlmannii* was used to treat skin infections in Livingstone. The* Lannea *species were used to treat skin diseases in South Africa [27]. Chinsembu and Hedimbi [17] found that* Lannea zastrowina* was used as a remedy for skin rashes and herpes zoster in Katima Mulilo in Namibia. Elsewhere,* Lannea* species were known to have antibacterial [29] and antiviral [30] properties, making them good candidates for treating microbial skin infections.

The plant* Kigelia africana*, used in this study to manage boils, was also used in Ghana to treat skin ailments including fungal infections, boils, psoriasis and eczema, leprosy, syphilis, and cancer [31]. The plant is known to contain iridoids which confer antibacterial properties [32].* Euclea divinorum* and* Ximenia americana,* skin remedies described in this study, were also documented as skin treatments by [27] in South Africa. Plants such as* Acacia, Kigelia africana,* and* Maytenus senegalensis* are used as ethnomedicine for skin infections [33].

Some of the plant taxa used to manage diarrhoea in this study have also been reported to treat diarrhoea in other studies.* Achyranthes aspera* L. is a known treatment for diarrhoea [34, 35];* Asparagus racemosus* roots have been used traditionally in Ayurveda for the treatment of diarrhoea and dysentery [33];* K. africana* is a known antidiarrhoeal remedy and* Parinari curatellifolia* attenuates diarrhoea [36, 37].

*Oncoba spinosa* is an antidote for diarrhoea in Ethiopia [38]. Studies in Nigeria showed that extracts of* Acacia nilotica* produced comparable antidiarrhoeal activity similar to loperamide, a drug widely employed against diarrhoeal disorders [39].* Garcinia livingstonei* was a remedy for diarrhoea in KwaZulu-Natal Province, South Africa [40]; a decoction from the roots* Combretum collinum* was drunk for the treatment of diarrhoea [41];* Bridelia cathartica*,* Flacourtia indica,* and* Kirkia acuminata* are prescriptions for diarrhoea in Zimbabwe [42, 43].

Members of the genus* Grewia *were used as a remedy for diarrhoea in Katima Mulilo [17] and* Mimosa pigra *was harvested to treat diarrhoea in Rundu [18]. Rakotomalala et al. [44] showed that* M. pigra* is rich in tryptophan, quercetin, and several phenolic compounds which confer antioxidant and anti-inflammatory properties.* Dalbergia melanoxylon* has antidiarrhoeal effects [45].

Studies in Tanzania found that* Indigofera colutea* has antimicrobial activities and hence can be used to manage diarrhoea.* Ficus capensis* has vibriocidal and antiamoebic actions [46] and therefore is used to treat diarrhoea in Lubumbashi in the neighbouring Democratic Republic of the Congo [47]. An anti-inflammatory bioflavonoid, gossypin, is found in* Hibiscus vitifolius*, a good remedy for diarrhoea [48].* Syzygium cordatum* Hochst, due to its antibacterial properties, is an antidiarrhoeal remedy in Swaziland [49].

Many plants used to treat STIs in Livingstone were also used to manage STIs in Sesheke District, Zambia [18]. This is because inhabitants of both Livingstone and Sesheke mainly belong to the Lozi ethnic group. Therefore, they tap into a similar ancestral vein of indigenous knowledge. For example, in both Livingstone and Sesheke, gonorrhoea was treated with a couple of species of the genera* Lannea, Combretum*,* Terminalia*,* Diospyros, Ximenia,* and* Ziziphus*.

The Lozi people of Sesheke used 52 plant species in 25 families and 43 genera to treat gonorrhoea, syphilis, chancroid, chlamydia, genital herpes, and anogenital warts. STIs were frequently managed using the following plants:* Terminalia sericea*,* Strychnos cocculoides*,* Ximenia caffra*,* Cassia abbreviata*,* Cassia occidentalis*,* Combretum hereroense*,* Combretum imberbe*,* Dichrostachys cinerea*,* Boscia albitrunca*,* Momordica balsamina,* and* Peltophorum africanum *[18].

*Ziziphus mauritiana*, also known as Masau in Nyanja, is a wild fruit plant very rich in vitamin C. It contains 20 to 30% sugar, up to 2.5% protein, and 12.8% carbohydrates. The plant is a remedy for STIs because aqueous extracts and powders have broad-spectrum antibacterial activity. Its extracts are also used as a dressing to prevent bacterial infections and to aid in wound healing during male circumcision among the Lunda and Luvale people of Zambia [50]. The anti-HIV plant* Ximenia americana* contains oleic, hexacos-17-enoic (ximenic), linoleic, linolenic, and stearic acids. Its oil consists of very long chain fatty acids with up to 40 carbon atoms.* X. americana* is also used to manage STIs including gonorrhoea in Western Province, Zambia [18].

*Euclea divinorum*, a treatment for gonorrhoea in Livingstone, had antibacterial action with minimum inhibitory concentration values ranging from 25.0 mg/mL to 0.8 mg/mL and moderate cytotoxicity [51].* Ximenia americana* and* Croton megalobotrys*, known as Mtswanza and Muchape (resp.) among the Kore-kore people of Chiawa District in Zambia, are also prepared as formulations for gonorrhoea [52].

Many species of* Acacia* are used to treat TB and pneumonia, owing to their antibacterial and anti-HIV activities [53, 54].* Acacia nilotica* leaf, bark and root ethanol, or ethyl acetate extracts were active against* Mycobacterium aurum*, MIC = 0.195–1.56 mg/mL [55].* Combretum imberbe* contains pentacyclic triterpenes, with MIC = 1.56–25 *μ*g/mL against* Mycobacterium fortuitum *[55].* Maytenus senegalensis* is a known anti-HIV and antimycobacterial treatment in Uganda and Tanzania [56, 57]. A* Cleome* species was used to treat TB in Livingstone. In South Africa, Hurinanthan [58] found that* Cleome monophylla *leaf extract had anti-HIV-1 reverse transcriptase activity.* Cleome gynandra *is a treatment for chancroid in Sesheke and a remedy for malaria in other parts of Zambia [17, 18].

Studies show that HIV/AIDS is associated with low libido in men, sometimes because of depression and poor moods [59, 60]. Men on ART were also associated with sexual dysfunction [61]. Unsurprisingly, loss of libido and erectile dysfunction in men were commonly associated with HIV infection in Livingstone. HIV-infected men suffering from loss of libido and erectile dysfunction often used herbs to restore their sexual prowess.* Mucuna pruriens*, a plant with antibacterial activity [62], is also known to improve fertility, sexual behaviour, and erectile function in animals [63–65]. Extracts of the plant* Hoslundia opposita* corrected erectile dysfunction in Livingstone men living with HIV infection and were also commonly used to manage noninsulin dependent diabetes mellitus in Tanzania [66]. Erectile dysfunction and loss of libido are common in men with diabetes [67].

## 5. Conclusions

In Livingstone, Southern Province, Zambia, traditional healers and other knowledge holders use 94 medicinal plant species to manage HIV/AIDS-related diseases mainly skin infections, diarrhoea, STIs, TB, cough, malaria, and oral infections. Majority of the plants belonged to the families Fabaceae and Combretaceae. Most plant leaves and roots were utilized to treat two or more disease conditions related to HIV infection. These multiuse medicinal plants probably contain broad-spectrum antimicrobial agents but may also face the threats of overharvesting, thus requiring special regulations for their genetic conservation.

The indigenous knowledge of medicinal plants is quite consistent especially for managing common HIV/AIDS-related conditions such as malaria, oral infections, fever, flu, colds, and headache. Although the results of this study are consistent with ethnobotanical and antimicrobial data from many reports in the literature, further studies are needed to confirm the antimicrobial efficacies, pharmacological, cytotoxicity, and active chemical ingredients of the plants.

## Figures and Tables

**Table 1:** Plants used to manage HIV/AIDS related diseases in Livingstone, Southern Province, Zambia.

Family	Botanical name	Vernacular name	Growth form	Frequency index, voucher number	Diseases treated	Plant parts used, preparation, and mode of administration
Acanthaceae	*Barleria kirkii* T. Anderson	Chavani	Herb	13.3, L144	HIV/AIDS	Leaf decoction is drank
Amaranthaceae	*Achyranthes aspera* L.	Tantajulo	Herb	20.0, L186	Cancer, pneumonia, cough, diarrhoea, fungal infections of the skin, genital warts	Root infusion or whole plant decoction is drank; paste of plant is applied to skin
Anacardiaceae	*Lannea stuhlmannii* (Engl.) Eyles	Mungangacha, Mucheche	Tree	83.3, L245	Gonorrhoea, syphilis, herpes zoster, herpes simplex, skin infections, HIV/AIDS	Crushed leaves or roots are boiled in water, decoction is drank; stem bark decoction is used to wash affected skin
Anacardiaceae	*Lannea discolor* (Sond.) Engl.	Mungongwa	Tree	50.0, L235	Diarrhoea, gonorrhoea	Fruit pulp eaten to relieve diarrhoea; root infusion drank to relieve gonorrhoea
Annonaceae	*Friesodielsia obovata* (Benth.) Verdc.	Muchinga	Shrub	20.0, L216	Skin rashes	Pounded leaves rubbed into skin
Annonaceae	*Artabotrys brachypetalus* Benth.	Mulandabala	Climber	13.3, L230	Skin infections	Crushed leaves rubbed into skin
Apiaceae	*Steganotaenia araliacea* Hochst.	Mupelewa	Tree	60.0, L113	Headache	Root decoction is drank
Arecaceae	*Hyphaene petersiana* Klotzsch ex Mart.	Kakunka, Mapokwe	Tree	20.0, L217	Malaria, cough, tuberculosis, skin rashes, sores related to STIs	Palm fruit is eaten raw or boiled to treat malaria and cough; seeds are used to treat TB; sap is applied to heal skin rashes and STIs
Asclepiadaceae	*Fockea angustifolia* K. Schum.	Mutindika, Nanyama	Herb	83.3, L210	Cough	Tuber is eaten raw
Asparagaceae	*Asparagus setaceus* (Kunth) Jessop	Mutandamyoba	Climber	50.0, L207	Eczema	Whole plant is crushed and rubbed into affected skin
Asparagaceae	*Sansevieria deserti* N. E. Br.	Musombo, Mukonje	Herb	13.3, L360	Oral infections	Leaves are chewed and then spitted
Asparagaceae	*Asparagus racemosus* Willd.	Mutandamyoba, Ilutwa	Climber	20.0, L433	Pneumonia, cough, diarrhoea, syphilis	Whole plant is boiled; decoction is drank
Asteraceae	*Vernonia amygdalina* Delile	Musoboyo	Shrub	83.3, L481	Coughs, tuberculosis, malaria	Leaves are boiled; decoction is drank
Bignoniaceae	*Kigelia africana* (Lam.) Benth.	Muzungula	Tree	83.3, L485	Syphilis, herpes simplex, diarrhoea, boils, colds, flu	Stem bark and leaves are boiled
Burseraceae	*Commiphora mollis* Engl.	Muntyokela	Tree	50.0, L312	Treats swollen pancreas in patients on antiretroviral therapy	Stem bark is crushed and boiled; decoction is drank
Capparaceae	*Boscia salicifolia* Oliv.	Mulaba, Kabombwe	Tree	70.0, L406	Syphilis, HIV/AIDS	Roots are ground and left in water; infusion is drank
Capparaceae	*Capparis tomentosa* Lam.	Chonswe	Shrub	20.0, L329	Syphilis rashes; HIV/AIDS; cryptococcal meningitis, oral candidiasis, herpes zoster, herpes simplex, chronic diarrhoea	Roots are crushed and boiled and decoction is drank; crushed leaves are applied to sores or soaked in water used to wash the mouth
Capparaceae	*Cleome hirta* Oliv.	Mulangazuba, Kalungukachisiungwa	Herb	13.3, L303	Pneumonia, tuberculosis, fungal infection of the skin, malaria	Leaf infusion is drunk
Celastraceae	*Maytenus senegalensis* (Lam.) Exell	Mukuba	Shrub	50.0, LV235	Tuberculosis	Leaves are crushed and soaked in water; infusion is drank
Celastraceae	*Hippocratea africana* Loes. ex Engl.	Mulele	Climber	60.0, LV280	Malaria	Root decoction is drunk
Chrysobalanaceae	*Parinari curatellifolia* Planch. ex Benth.	Mubulabula, Mula	Tree	13.3, LV245	Toothache, diarrhoea	Fruit eaten raw
Clusiaceae	*Garcinia livingstonei* T. Anderson	Mutungwa, Mukwanaga	Tree	83.3, LV226	Cryptococcal meningitis, herpes zoster, herpes simplex, skin rashes Tuberculosis chronic diarrhoea	Fruit eaten raw or in porridge
Combretaceae	*Combretum collinum* Fresen.	Mukunza, Mulamana	Tree	43.3, LV187	Chronic diarrhoea, tuberculosis, cough	Roots are crushed and soaked in water overnight; filtrate is drank
Combretaceae	*Combretum imberbe* Wawra	Mubimba, Muzwili	Tree	83.3, LV238	General STIs, tuberculosis	Stem bark is crushed and soaked in water overnight; filtrate is drank
Combretaceae	*Combretum apiculatum* Sond.	Mukalanga, Kalanga, Nkalanga	Tree	50.0, LV220	General STI syndromes; tuberculosis	Stem bark is crushed and soaked in water overnight; filtrate is drank
Combretaceae	*Combretum elaeagnoides* Klotzsch	Mukalanga, Mukupo	Tree	30.0, L576	Malaria, tuberculosis, diarrhoea	Fresh leaves are crushed and soaked in water overnight; filtrate is drank
Combretaceae	*Combretum hereroense* Schinz	Namazubo	Tree	53.3, L437	Gonorrhoea	Roots are crushed and soaked in water overnight; filtrate is drank
Combretaceae	*Terminalia prunioides* M. A. Lawson	Mutala, Mukonono, Mulumbu	Tree	83.3, L247	Gonorrhoea, syphilis, HIV/AIDS	Outer parts of roots are dried, crushed, and mixed with water overnight; infusion is drank
Combretaceae	*Combretum mossambicense* Engl.	Numinambelele, Silutombolwa, Mutombololo	Climber	36.7, LV475	Gonorrhoea, syphilis	Fresh leaves are crushed and soaked in water overnight; filtrate is taken orally
Combretaceae	*Combretum paniculatum* Vent.	Mutombolo	Shrub	50.0, L322	Malaria, diarrhoea	Leaves are pounded and soaked in water; infusion is drank
Connaraceae	*Byrsocarpus orientalis* Baill.	Kazingini	Shrub	13.3, L521	Skin abscesses and boils	Young stems are cut and boiled, and decoction is applied to skin
Dioscoreaceae	*Dioscorea cochleari-apiculata* De Wild.	Mpama	Climber	13.3, L511	Syphilitic sores, chancroid	Root decoction drank
Ebenaceae	*Diospyros mespiliformis* Hochst. ex A.DC.	Mchenja	Tree	83.3, L109	Malaria	Crushed roots are boiled; filtrate is drank
Ebenaceae	*Diospyros quiloensis* (Hiern) F. White	Musiaabwele	Tree	50.0, LV339	Gonorrhoea, syphilis, malaria	Crushed stem bark is boiled; filtrate is drank
Ebenaceae	*Euclea divinorum* Hiern	Munyansyabweli	Tree	20.0, LV140	Syphilis, gonorrhoea, genital herpes, oral candidiasis, abscesses, diarrhoea	Roots are ground and boiled in water and decoction is taken orally
Erythroxylaceae	*Erythroxylum zambesiacum* N. Robson	Mubalubalu	Tree	16.7, LV277	Malaria, headache	Roots are cut into small pieces and boiled, and decoction is administered orally
Euphorbiaceae	*Croton gratissimus* Burch.	Mungai, Kanunkila Mpati	Tree	66.7, LV399	Syphilis	Stem bark is boiled; decoction is drank and used to wash sores
Euphorbiaceae	*Croton megalobotrys* Müll. Arg.	Mutua, Mutuatua	Tree	60.0, S149	Gonorrhoea	Leaves are boiled and decoction is drank
Euphorbiaceae	*Pseudolachnostylis maprouneifolia* Pax	Mukunyu	Tree	23.3, S108	Diarrhoea, pneumonia	Stem bark decoction is drank
Euphorbiaceae	*Bridelia cathartica* G. Bertol.	Munyanyamenda	Shrub	13.3, S154	Oral infections, diarrhoea, gonorrhoea, malaria	Leaves and fruits are chewed raw to act as mouthwash; stem bark infusion is drank
Euphorbiaceae	*Margaritaria discoidea* (Baill.) G. L. Webster	Mulyankanga	Shrub	16.7, S119	Skin rashes and sores; headache	Stem bark
Euphorbiaceae	*Phyllanthus reticulatus* Lodd.	Mwichechele	Shrub	26.7, S260	Herpes simplex	Crushed leaves are rubbed to affected areas of skin
Fabaceae	*Acacia albida* Delile	Musangu, Muunga	Tree	50.0, S100	Syphilis sores, herpes zoster	Crushed leaves and stem bark are applied to heal sores
Fabaceae	*Acacia nigrescens* Oliv.	Mwabaa, Mukwena	Tree	60.0, S78	Herpes zoster	Leaves and stem bark are boiled, decoction used to
Fabaceae	*Acacia polyacantha* Willd.	Mumbu, Mukotokoto, Luntwele	Tree	50.0, S63	Gonorrhoea, herpes zoster	Leaves and stem bark are crushed and boiled; decoction is drank or used to wash affected skin
Fabaceae	*Afzelia quanzensis* Welw.	Mupapa, Mukamba	Tree	76.7, S27	General STIs	Leaves are pounded and soaked in water overnight and then drank
Fabaceae	*Albizia amara* (Roxb.) Boivin	Kankumbwila,Mukangola	Tree	60.0, S44	Gonorrhoea, diarrhoea	Stem bark infusion is drank
Fabaceae	*Lonchocarpus capassa* Rolfe	Mukololo	Tree	13.3, S31	Gonorrhoea, cough	Stem bark and leaves are mixed, crushed, soaked in water, and filtered and infusion is drank
Fabaceae	*Peltophorum africanum* Sond.	Muzenzenze	Tree	83.3, K203	General STIs, oral infections	Roots are soaked in water and infusion is drank to treat STIs; leaves are crushed and soaked in water; infusion is used to wash mouth
Fabaceae	*Pterocarpus antunesii* Harms	Mukambo	Tree	26.7, K166	Diarrhoea	Stem bark infusion is drank
Fabaceae	*Acacia goetzei* Harms	Mwaba	Tree	46.7, K88	Cough, pneumonia	Root decoction is drank
Fabaceae	*Acacia nilotica* Delile	Mukoka	Tree	66.7, K56	Tuberculosis, diarrhoea, gonorrhoea, dental caries	Twigs used as chewing sticks to treat dental caries
Fabaceae	*Cassia abbreviata* Oliv.	Mululwe	Tree	83.3, K39	Gonorrhoea, diarrhoea	Root infusion is drank
Fabaceae	*Dalbergia melanoxylon* Guill. & Perr.	Musonkomo	Shrub	13.3, K17	Diarrhoea	Root decoction is drank
Fabaceae	*Piliostigma thonningii* (Schumach.) Milne-Redh.	Musekese	Tree	60.0, K12	Coughs, skin rashes, gonorrhoea, syphilis	Stem bark and roots are boiled; decoction is drank to heal coughs and STIs; leaf infusion is used to wash infected skin
Fabaceae	*Acacia ataxacantha* DC.	Lubamfwa	Shrub	50.0, K60	Gonorrhoea, syphilis	Roots are boiled and decoction is drank
Fabaceae	*Acacia schweinfurthii* Brenan & Exell	Lubua, Mokoka	Shrub	33.3, L34	Gonorrhoea, syphilis	Roots, stem bark, and leaves are mixed, pounded, and soaked in water and infusion is drank
Fabaceae	*Dichrostachys cinerea* (L.) Wight & Arn.	Katenge, Mugee	Shrub	66.7, L79	Gonorrhoea, syphilis; oral candidiasis, and skin rashes	Stem bark is boiled and decoction is drank, used to wash oral cavity, or used to disinfect skin by washing
Fabaceae	*Mimosa pigra* L.	Muchabachaba, Sichatubabi	Shrubs	83.3, L56	Diarrhoea, genital ulcers, gonorrhoea	Leaves and small and young stems are dried, pounded into powder, mixed with water, and filtered and extract is drank or used to wash genital ulcers
Fabaceae	*Sesbania sesban* Britton	Mbelembele	Shrub	13.3, L77	Malaria	Roots are boiled and decoction is administered orally
Fabaceae	*Indigofera colutea* (Burm. f.) Merr.	Kapalupalu	Herb	13.3, L59	Diarrhoea	Whole plant is pounded, soaked in water overnight, and filtered, and infusion is drank to alleviate diarrhoea
Fabaceae	*Abrus precatorius* L.	Musolosolo	Climber	60.0, KC280	Gonorrhoea, syphilitic ulcers, genital herpes; oral candidiasis, ulcer boils	Whole plant is crushed, boiled in water, and filtered; decoction is drank; crushed leaves soaked in water and used to wash mouth and syphilitic ulcers
Fabaceae	*Mucuna pruriens* (L.) DC.	Muyuyu	Climber	20.0, KC245	Weight loss, lack of libido in men	Pods and beans are boiled and consumed for body building and to act as an aphrodisiac
Flacourtiaceae	*Flacourtia indica* (Burm. f.) Merr.	Mutumbula	Shrub	13.3, KC350	Diarrhoea	Leaves chewed raw
Flacourtiaceae	*Oncoba spinosa* Forssk.	Mukumbuzu	Shrub	16.7, KC2	Dysentery	Roots and fruits are boiled and decoction is drunk
Kirkiaceae	*Kirkia acuminata* Oliv.	Musanta, Muzumina	Tree	13.3, KC18	Diarrhoea	Stem bark infusion is drank
Lamiaceae	*Vitex payos* (Lour.) Merr.	Mfudu, Muyankonga	Tree	30.0, KC36	Coughs	Fruit eaten raw or leaves burnt and smoke inhaled as cough medicine
Lamiaceae	*Hoslundia opposita* Vahl	Musombwani	Shrub	60.0, KC48	Coughs, flu, fever, loss of libido in men; skin wounds	Roots are boiled and decoction is drunk; sugary fruits are eaten raw to boost libido; crushed leaves are rubbed into skin to heal wounds
Lamiaceae	*Premna senensis* Klotzsch	Mumpika	Shrub	13.3, KC360	Syphilitic sores, skin ulcers	Leaf infusions applied to sores
Lamiaceae	*Vitex petersiana* Klotzsch	Mufulibulimbo, Mukoma	Shrub	23.3, KC312	Gonorrhoea	Leaf decoction is drank to treat gonorrhoea;
Lamiaceae	*Clerodendrum capitatum* (Willd.) Schumach.	Shamanya	Herb	60.0, L402	Gonorrhoea, erectile dysfunction, pneumonia, diarrhoea	Leaf and root decoctions drunk
Loganiaceae	*Strychnos potatorum* L. f.	Musisilombe	Tree	40.0, KC464	Syphilis	Infusions of leaves are drank
Loganiaceae	*Strychnos innocua* Delile	Mutimi, Muhuluhulu, Mwabo	Tree	20.0, KC216	Gonorrhoea, sore throat	Eat fruit pulp
Malvaceae	*Azanza garckeana* (F. Hoffm.) Exell & Hillc.	Makole, Munego	Tree	60.0, KC143	Malaria	Eat raw fruit, cook, and eat as relish
Malvaceae	*Sida alba* L.	Mulyangombe, Babani	Shrub	20.0, KC525	Gonorrhoea	Roots and leaves are boiled; decoction is drank
Malvaceae	*Hibiscus vitifolius* L.	Mubaluba	Herb		Chronic diarrhoea	Leaves are boiled and filtered through wire sieve and decoction is drank
Meliaceae	*Khaya nyasica *Stapf ex Baker f.	Mululu	Tree	43.3, KC113	Fever	Stem bark infusion is drank
Meliaceae	*Trichilia emetica* Vahl	Musikili	Tree	56.7, KC217	Fever, pneumonia; skin rashes	Root decoction is drunk; leaves rubbed onto skin
Menispermaceae	*Cissampelos mucronata* A. Rich.	Itende	Climber	73.3, KC210	Syphilis, chancroid	Whole plant is cut into small pieces and boiled and decoction is drank after filtering
Moraceae	*Ficus capensis* Hort. Berol. ex Kunth & C. D. Bouché	Mukuyu	Tree	60.0, KC207	Diarrhoea, tuberculosis, skin sores, genital warts	Fresh leaves are boiled in water and decoction is drank or used to wash warts and skin sores
Moraceae	*Ficus sycomorus* L.	Mukuyu	Tree	76.7, KC360	Cough, tuberculosis, periodontitis, and oral candidiasis	Fresh leaves are boiled in water and decoction is drank or used to wash the mouth
Myrtaceae	*Syzygium cordatum* Hochst.	Katope	Tree	83.3, KC433	Diarrhoea	Stem bark decoction is drunk
Myrtaceae	*Syzygium guineense* DC.	Mutoya, Katope	Tree	83.3, KC481	Abscesses, skin rashes, diarrhoea	Fruits eaten raw; stem decoction applied to affected skin
Olacaceae	*Ximenia americana* L.	Munchovwa, Muchonfwa	Tree	83.3, KC318	Candidiasis, malaria, throat infection, tonsillitis, gonorrhoea, diarrhoea, skin rashes	Roots, leaves, and fruits are crushed while fresh and mixed with water overnight and taken orally
Pedaliaceae	*Sesamum angolense* Welw.	Bwengo	Herb	13.3, KC267	Skin rashes	Leaves are crushed and used as soap to bath skin
Plumbaginaceae	*Plumbago zeylanica* L.	Sikalutenta	Shrub	83.3, KC318	Generally treats all STI symptoms	Root infusion is drank
Ranunculaceae	*Clematis brachiata* Ker Gawl.	Kalatongo	Climber	20.0, KC354	Coughs, headache, fever	Leaves are boiled and drunk as a tea; sometimes honey is added as a sweetener
Rhamnaceae	*Berchemia discolor* (Klotzsch) Hemsl.	Mwiiyi, Mwinji	Tree	60.0, KC235	Cough	Use fruit in porridge
Rhamnaceae	*Ziziphus mauritiana* Lam.	Masawu, Masawu	Tree	40.0, KC226	Gonorrhoea, syphilis	Fruit eaten raw, apply to wound, and put into porridge
Rhamnaceae	*Ziziphus mucronata* Willd.	Muchechete, Mwichechete	Tree	83.3, KC144	Gonorrhoea, syphilis, boils, pneumonia, cough	Root or leaf infusion is drank
Sapotaceae	*Mimusops zeyheri* Sond.	Mukalanjoni	Tree	40.0, KC120	Oral candidiasis	Fruit is eaten or root infusion is used as a mouthwash
Tiliaceae	*Grewia flavescens* Juss.	Namulomo, Mukunyukunyu	Shrub	40.0, KC199	Diarrhoea	Eat fruit pulp to relieve diarrhoea
Tiliaceae	*Corchorus tridens* L.	Delele	Herb	13.3, KC101	Syphilitic ulcers, chancroid	Root infusion drank or applied to ulcers
Vitaceae	*Cissus quadrangularis* L.	Mulungalunga, Chamulungelunge	Climber	40.0, KC78	Malaria, gonorrhoea	Sap is drank; whole plant infusion is drank

**Table 2:** Informant consensus factor (*F*_IC_) for different ailments.

Ailment	Number of species	Number of citations	*F*_IC_
Diarrhoea/dysentery	33	120	0.73
Skin infections	36	108	0.67
STIs	70	210	0.67
Malaria	15	77	0.82
TB/pneumonia	19	68	0.73
Oral infections	11	55	0.81
Cough	18	71	0.76
Fever/flu/colds/headache	10	45	0.80
Libido/erectile dysfunction	4	12	0.73
Meningitis	2	6	0.80
Weight loss	1	4	1.00
Cancer	1	4	1.00
